# Checklist of British and Irish Hymenoptera - Introduction

**DOI:** 10.3897/BDJ.2.e1113

**Published:** 2014-06-17

**Authors:** Gavin R. Broad

**Affiliations:** † The Natural History Museum, London, United Kingdom

## Introduction

The last complete checklist of the British and Irish Hymenoptera was that of Fitton et al. (1978). Much has changed in the intervening 36 years, including many changes to the higher classification of Hymenoptera and, of course, the discovery of many additional species. In producing this checklist we (the authors of the various chapters) have tried to provide a useful entry to the literature dealing with the identification and classification of the species involved, particularly for the less familiar parasitoid groups. The checklist also begins the process of summarising data on species' distribution on a country-level basis. I hope that this will serve to stimulate increased interest in this relatively neglected part of the British and Irish fauna.

This volume serves as a standard reference point for the British and Irish Hymenoptera fauna, and, hopefully, provides a backbone to recording efforts and the underlying taxonomy. Having an endpoint to this project has stimulated much research in to our fauna, including the critical examination of museum collections, and thus pushed some checklist sections to a state they might not otherwise have reached. Of course, a checklist is never finished. The next incarnation will be digital, and the checklist is already in the process of migrating to a Scratchpad (Hymenoptera of the British Isles), where updates will be maintained as and when changes occur.

### Criteria for inclusion

Additions to the British and Irish list since Fitton et al. (1978) are referenced (with a note 'added by...'). Species have been included that have not previously been formally published as being British or Irish but which are represented by reliably identified specimens in collections. For these species, collection depositories are listed so that the specimens can be traced. Known introductions, occasional or otherwise, including those species that are only able to sustain populations indoors, are also included, but noted as introduced. However, introduced or adventive species are not included in the counts of valid British and Irish species (Table 1).

Our knowledge of the distribution of most Hymenoptera is extremely rudimentary. Where practicable, country-level occurrences are listed. The recording units are England, Ireland, Isle of Man, Scotland and Wales. Data on the Channel Islands are not included as they are faunistically (and geographically) much closer to France. Thus, species known only from the Channel Islands within the UK, and covered by handbooks such as Richards (1980), are excluded from the checklist. Distribution data have been harvested from many publications, with citations provided for each family. For some groups, particularly the Ichneumonoidea and Chalcidoidea, much of the distribution data have been obtained from systematically checking through the collections of the Natural History Museum, London (NHM), and (for Ichneumonoidea) National Museums of Scotland, Edinburgh (NMS). The collections of the NMS have been invaluable in updating this checklist as Mark Shaw has very actively encouraged taxonomists to use the collections, which contain a large amount of recently collected, often reared, material. Additional valuable distributional and biological data will be contained within the collections of other museums and for other taxa but time and available expertise are constrained. The aculeate superfamilies are much better recorded with distribution data being gathered by the Bees, Wasps and Ants Recording Society and published in a series of atlases (Edwards 1997, Edwards 1998, Edwards and Telfer 2001, Edwards and Telfer 2002, Edwards and Broad 2005, Edwards and Broad 2006, Edwards and Roy 2009, Collins and Roy 2012). These distribution data and maps are now available online (www.bwars.com). Another large group of Hymenoptera, the sawflies, is also covered by an active group of recorders (the Sawfly Study Group) and there is a Nocturnal Ichneumonoidea Recording Scheme (http://www.nhm.ac.uk/research-curation/about-science/staff-directory/life-sciences/g-broad/index.html). Hopefully, recording efforts for parasitoids will gather pace and distribution data will begin to bear more of a resemblance to the real distribution of a species but, for the moment, the majority of British parasitoid Hymenoptera are known from small numbers of specimens in collections.

### Taxonomy

This is not a catalogue but, nevertheless, we have generally included extensive synonymy and notes on the sources of taxonomic changes, which seemed essential given the shortage of readily accessible resources on the Hymenoptera as a whole. Where there is an extensive British literature, which is mainly true of the sawflies and the aculeates, synonyms have mostly been restricted to those names that have appeared in the British literature. For most other groups there is little in the way of a body of British literature. In these cases, all (or at least all of the Palaearctic) synonyms are listed. Recent catalogues have been used and cited, where such works exist. Deviations from these works, usually from more recent publications, are cited. We have not always followed the most recent taxonomic changes when we have considered these to be incorrect or poorly justified.

Species names are listed in their current combinations followed by the original combination; synonyms follow the format, '*name* (author, date, *original combination*) [when the combination has since changed]'. Due to the nature of the listing of names in a checklist, some synonymous names appear here in combinations (and hence sometimes specific name endings) that may not have appeared in the literature before. It was felt that this was a better solution than listing all names in their original combinations, which in many cases will hardly ever have been used in the recent literature. Hopefully all specific endings are correct but some errors may well have slipped through the net. A checklist is full of compromises but our guiding intention has been to present all the relevant information in a manner that it as compact and informative as possible.

Subspecific names are not listed in the main body of the checklist. Subspecific names in Hymenoptera, as in many insect groups, are inconsistently applied and, where they are applied, it is generally with very little justification. Where subspecific names have been treated recently as synonymous they are simply listed in the synonymy. Where subspecies have been used as valid taxonomic ranks in recent publications an explanatory footnote has been added.

In a few cases, names have been included in the format 'sp.W', in anticipation of a forthcoming description of a new species. Varietal names have generally been excluded, except where they have been made available as species-group names under article 45.6 of the ICZN, or when they were described on the basis of British specimens. Sometimes it is unclear whether a varietal name is valid or not and we have included these.

### Size of the British and Irish fauna

At 7,761 species, the Hymenoptera is now comfortably the largest insect order in the British Isles. That will come as no surprise to those of us who work on them but the disparity between the size of the Hymenoptera fauna and that of the Coleoptera or Lepidoptera may surprise some entomologists. That some sections of this checklist now differ substantially from their 1978 versions is a sign of the progress that has been made; that the checklists of some fairly large families remain very little changed since 1978 is an indication that there is still much work to be done in assessing the true scale of the British fauna. It needs to be emphasised that our knowledge of some families is still rudimentary. For example, the checklist of British and Irish Eurytomidae is still based on a few papers by Claridge and co-workers and on Walker's original descriptions. Similarly, the amount of 'added value' that could be brought to sections of the checklist was highly variable as there was no taxonomic expertise available for several groups; this is particularly true of the family Ceraphronidae. Whilst much unpublished information was added to the Ichneumonoidea checklist, for example, and the literature rather thoroughly surveyed, the same could not be achieved for the Ceraphronidae section, because that knowledge base is simply not there.

In deriving the total species numbers in Table 1, names listed with question marks (usually uncertain identifications) are not listed, nor are those known only from introductions where the species is established only in artificial, ameliorated climates, such as indoors or in greenhouses. The comparisons with the 1978 totals require some caution as some names in the previous checklist have been synonymised. A comparison of the species totals between the two checklists, accounting for the various fates of names, will be published separately; the figures provided in Table 1 are simply a raw comparison that gives an idea of the increase in knowledge in the intervening years. The relationship between total number of known British and Irish species and percentage increase across the recording period give some idea of the amount of study those groups have received. For a very few families, particularly Vespidae, the relative change reflects the level of actual change in the fauna, with colonisation of Britain by several species. Generally though, the recorded level of change is indicative of the amount of work on that group.

Fig. 1

### Higher level classification

Traditionally the Hymenoptera has been divided into three sub-orders, the 'Symphyta' for the sawflies, 'Parasitica' for the non-aculeate parasitoid wasps and the 'Aculeata' for the aculeates, i.e., the group of ants, bees and wasps that has lost the ability to use the sting as an ovipositor. Although the 'Parasitica' and 'Aculeata' together form a clade, the Apocrita, and the aculeates form a monophyletic group within the Apocrita, 'Symphyta' and 'Parasitica' are each demonstrably paraphyletic (e.g., Vilhelmsen 1997, Schulmeister et al. 2002, Heraty et al. 2011) so we do not employ sub-order ranks for these assemblages. However, we have grouped together these three assemblages of superfamilies because each of these groups, in Britain at least, tend to be worked on by distinct communities of recorders and biologists. Similarly, we have grouped the bees together (as the series 'apiformes') rather than simply listing all of the families of Apoidea alphabetically (as the digger wasp families form a basal grade with the bees nested within, e.g., Melo 1999). Otherwise, the checklist is alphabetical. It is often the practice to order taxa within a phyletic sequence, or natural classification, reflecting the phylogeny of the group. We have not opted for this approach for three reasons: (1) it is impossible to reflect a phylogenetic topology in a linear sequence, which has the effect of encouraging the view that those taxa towards the end are the most 'advanced', possibly projecting the idea of an evolutionary trajectory in users' minds (as in all those representations of the tree of life that feature *Homo sapiens* at the apex); (2) we know too little about the inter-relationships of higher taxa within the Hymenoptera and next to nothing about the inter-generic or species-level relationships of the vast majority of Hymenoptera; (3) when alphabetically arranged, it is much simpler to find a name.

The superfamily classification (Table 1) basically follows Sharkey (2007), whose summary of hymenopteran relationships is derived from works such as Vilhelmsen (1997), Vilhelmsen (2001), Schulmeister et al. (2002), Dowton and Austin (2001) and others. The study of Klopfstein et al. (2013) was timely, based on a larger and more complete molecular and morphological dataset than any previous attempt at reconstructing the Hymenoptera tree of life. The conclusions generally support the superfamily classification as summarised by Sharkey (2007), but highlight several areas of uncertainty and likely future change, when further data, based on additional taxa, are gathered. The Proctrotrupoidea has been successively chopped up since 1978 and I follow Sharkey (2007) and Klopfstein et al. (2013) in further trimming the Proctotrupoidea by treating the Diaprioidea as a separate superfamily. Note that, although there are only two British families of Proctotrupoidea and one of Diaprioidea, these are more diverse in the wider world, with several extralimital families. However, Klopfstein et al. (2013) found that the Diaprioidea may be paraphyletic with respect to the Chalcidoidea and Mymarommatoidea. Recent studies have differed in suggesting that Ismarinae may (Klopfstein et al. 2013) or maynot (Heraty et al. 2011) belong in the Diapriidae. The family-level classification of Chalcidoidea is particularly poorly supported and there is a strong possibility of further changes inthe near future; Heraty et al. (2013) have recently made some changes to family-level classification of Chalcidoidea and highlighted remaining areas of uncertainty. Neither the Vespoidea nor Tiphiidae is likely to be monophyletic (Pilgrim et al. (2008), Klopfstein et al. (2013)).

There are two approaches to the classification of bees, with some authors recognising a single family, Apidae, for this distinctive, monophyletic group (e.g., Fauna Europaea: http://www.faunaeur.org/), and other authors recognising a series of families (six in Britain). The first approach derives from the fact that bees are but one lineage of apoid wasps, equivalent to the Crabronidae or Sphecidae. The second approach emphasises the biological and phylogenetic distinctiveness of several clades of bees. I have followed the second approach, of dividing bees into several families, on the pragmatic grounds that this is the approach taken by Michener (2007) in his authoritative treatise on bee classification. The sources of nomenclature and classification are cited at the beginning of each higher taxon.

The family-level taxonomic ranks used here are Superfamily, Family, Subfamily and Tribe. Sub-tribes have not been included here; the use of sub-tribes is very inconsistent in Hymenoptera and they have generally been very poorly defined.

### Conventions and abbreviations

Various abbreviations are used in the checklist, relating to distribution, status, specimen depositories and nomenclature. Fuller explanations of nomenclatural terms and how they relate to the articles of the zoological code of nomenclature can be found in the International Code of Zoological Nomenclature (ICZN 1999 and online at http://iczn.org/node/40200).

Valid family, genus and species names are in bold type, synonymous names are indented and in regular type, listed in chronological order.

Superfamily **ICHNEUMONOIDEA**

Family **Ichneumonidae**

Subfamily CRYPTINAE

Tribe CRYPTINI

[***species***] taxon deleted from the British and Irish list

NHM Natural History Museum, London

NMS National Museums of Scotland, Edinburgh

UM Ulster Museum [J. Brock collection]

# known introductions occurring only under artificial conditions

? status (including uncertain synonymy) or identification in the British Isles uncertain

misident. has been misidentified as this name

nom. dub. *nomen dubium*, a name of doubtful status

nom. ob. *nomen oblitum*, ‘forgotten name’, does not have priority over a younger name

nom. nov. *nomen novum*, a replacement name

nom. nud. *nomen nudum*, an unavailable name, with no type specimen

preocc. name preoccupied (junior homonym)

stat. rev. *status revocatus*, revived status (e.g., raised from synonymy)

unavailable name unavailable under provisions of the ICZN code

var. variety, only available as a valid name under certain provisions of the ICZN code

-a- name based on agamic (asexual) generation (used in Cynipidae)

-s- name based on sexual generation (used in Cynipidae)

## Figures and Tables

**Figure 1.**
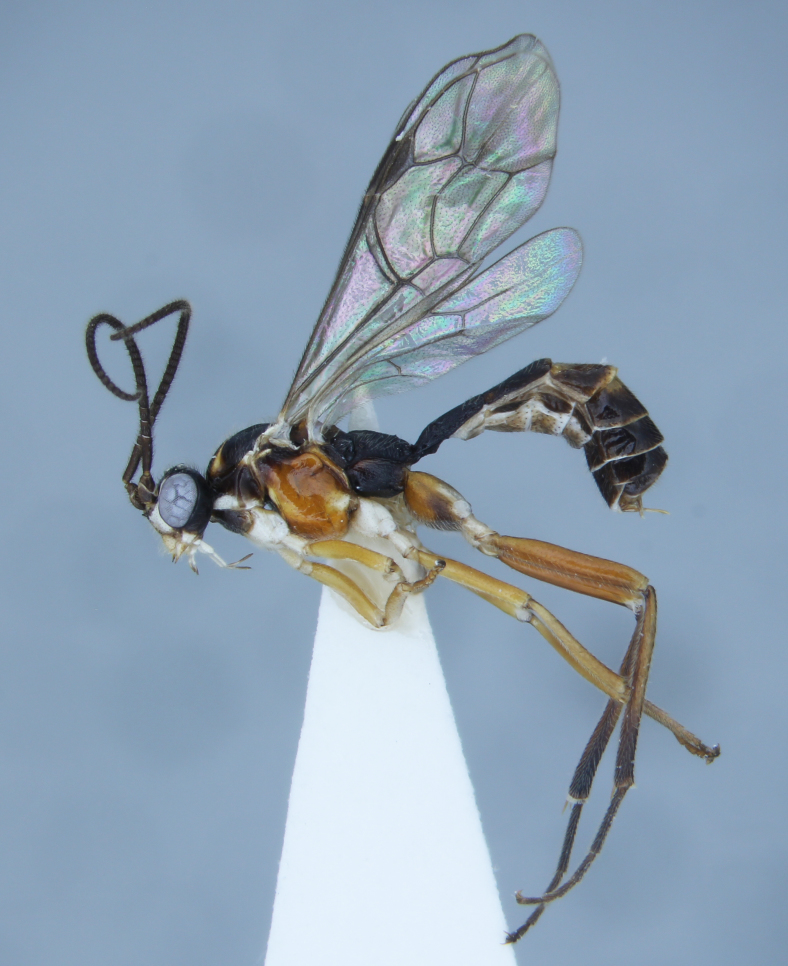
*Megastylus suecicus* van Rossem, one of 219 species of Ichneumonidae reported here as British for the first time.

**Table 1.** Classification of Hymenoptera adopted in this checklist, with numbers of confirmed British and Irish species in the 1978 and current checklists (not including known introductions that have failed to establish in the wild). Note that the spelling of Trigonalidae and Trigonaloidea, as opposed to 'Trigonalyidae', follows Aguiar et al. (2013).

**Series**	**Superfamily**	**Family**	**1978**	**2014**
sawflies	Cephoidea	Cephidae	12	13
	[Orussoidea	Orussidae	1?	1?]
	[Pamphilioidea	Megalodontesidae	3?	1?]
		Pamphiliidae	19	20
	Siricoidea	Siricidae	6	6
	Tenthredinoidea	Argidae	15	19
		Blasticotomidae	1	1
		Cimbicidae	15	17
		Diprionidae	9	9
		Tenthredinidae	390	446
	Xiphydrioidea	Xiphydriidae	2	3
	Xyeloidea	Xyelidae	2	3
parasitoids	Ceraphronoidea	Ceraphronidae	26	28
		Megaspilidae	65	64
	Chalcidoidea	Aphelinidae	35	39
		Azotidae	1	1
		Chalcididae	6	10
		Encyrtidae	191	221
		Eucharitidae	1	1
		Eulophidae	384	508
		Eupelmidae	13	15
		Eurytomidae	90	96
		Mymaridae	82	95
		Ormyridae	3	4
		Perilampidae	7	9
		Pteromalidae	523	567
		Signiphoridae	2	2
		Tetracampidae	7	8
		Torymidae	68	104
		Trichogrammatidae	27	37
	Cynipoidea	Cynipidae	79	86
		Figitidae	123	128
		Ibaliidae	1	2
	Diaprioidea	Diapriidae	295	276
	Evanioidea	Aulacidae	1	1
		Evaniidae	1	1
		Gasteruptiidae	5	5
	Ichneumonoidea	Braconidae	1149	1335
		Ichneumonidae	2003	2578
	Mymarommatoidea	Mymarommatidae	1	1
	Platygastroidea	Platygastridae	259	362
	Proctotrupoidea	Heloridae	3	3
		Proctotrupidae	30	39
	Trigonaloidea	Trigonalidae	1	1
	Apoidea - 'spheciformes' wasps	Crabronidae	109	121
		Sphecidae	4	4
	Apoidea - bees	Andrenidae	67	68
		Apidae	65	69
		Colletidae	20	21
		Halictidae	57	57
		Megachilidae	34	39
		Melittidae	6	6
	Chrysidoidea	Bethylidae	13	13
		Chrysididae	31	31
		Dryinidae	44	35
		Embolemidae	2	1
	Vespoidea	Formicidae	42	51
		Mutillidae	3	3
		Pompilidae	41	43
		Sapygidae	2	2
		Tiphiidae	3	3
		Vespidae	30	33
**Totals**	**20**	**62**	**6526**	**7764**
